# Understanding myocardial infarction

**DOI:** 10.12688/f1000research.15096.1

**Published:** 2018-09-03

**Authors:** Moussa Saleh, John A Ambrose

**Affiliations:** 1 Division of Cardiology, Department of Internal Medicine, University of California San Francisco-Fresno, 2335 E. Kashian Lane, Suite 460, Fresno, CA 97301, USA

**Keywords:** Myocardial Infarction, CAD, cardiogenic shock

## Abstract

Over the last 40 years, our understanding of the pathogenesis of myocardial infarction has evolved and allowed new treatment strategies that have greatly improved survival. Over the years, there has been a radical shift in therapy from passive healing of the infarction through weeks of bed rest to early discharge usually within 2 to 3 days as a result of immediate reperfusion strategies and other guideline-directed medical therapies. Nevertheless, challenges remain. Patients who develop cardiogenic shock still face a high 30-day mortality of at least 40%. Perhaps even more important is how do we identify and prevent patients from developing myocardial infarction in the first place? This article discusses these milestones of therapy and considers important issues for progress in the future.

## Introduction

Our understanding of the causes, diagnosis, and treatment of acute myocardial infarction (AMI) has evolved significantly over the last 40 years. In the early 20th century, AMI was generally considered a fatal event diagnosed only at autopsy. Until the 1970s, with appropriate understanding of its usual clinical presentation and diagnosis, it was conservatively managed with prolonged bed rest and afterwards with a sedentary lifestyle. Since then, there has been an explosion of information which has changed our understanding of its pathogenesis and markedly altered our treatment options, leading to vastly improved outcomes. This article will review where we came from and what our current understanding and management of this important condition are. We will also explore future treatment options.

## Definitions

AMI, usually referred to in lay terms as a heart attack, is most often caused by a decrease or stoppage of blood flow to a portion of the heart, leading to necrosis of heart muscle. This is generally the result of a blood clot in the epicardial artery that supplies that territory of heart muscle. It is now recognized that, based on how AMI is defined, not all cases necessarily require a blood clot etiologically. In all living tissue such as heart muscle, the blood supply must equal the oxygen demands of the muscle. This is termed the supply–demand ratio. It is now appreciated that an imbalance in this ratio (too little supply or too much demand) as might occur with a very rapid heart rate (too much demand) or a drop in blood pressure (too little supply) may lead to myocardial damage without the presence of a blood clot per se. Over the last 10 years, a universal definition of AMI has been available to help the clinician with its diagnosis ^1, 2^. This definition states that there must be a rise or fall (or both) in a blood test sensitive to heart muscle damage (troponin I or T) with at least one value above the 99th percentile of the upper reference limit along with clinical evidence for a diagnosis of AMI. This clinical evidence includes symptoms of ischemia, which include either electrocardiographic evidence indicative of ischemia such as ST segment changes or new left bundle branch block, development of pathological Q waves on electrocardiogram (ECG), or new wall motion abnormalities on cardiac testing or a combination of these.

## Nomenclature for myocardial infarction

Since the 1970s, the nomenclature defining myocardial infarction (MI) has changed several times. During the 1960s and 1970s, MI was characterized as transmural MI versus non-transmural MI. In transmural MI, the ischemia and injury affected the entire thickness of the myocardial muscle (endocardium, myocardium, and epicardium). This typically was the result of a complete occlusion of a large epicardial coronary artery by a thrombus resulting in decreased blood supply to all three layers of the heart muscle. On the other hand, non-transmural MI was defined as ischemia and injury that did not affect all three layers of the heart muscle, typically sparing the epicardium. This was considered to result from a significant decrease in blood supply to the territory with or without complete occlusion of a coronary artery or branch. In the 1980s, the nomenclature changed to include ECG evidence of MI. Q wave MI substituted for the old transmural MI definition and was used for an MI that involved all three layers of the heart muscle and hence would show a pathologic Q wave on ECG in two contiguous leads. Non-transmural MI became known as non-Q wave MI, as it was postulated that Q waves would not show up on ECG unless the entire thickness of the heart muscle was involved. However, over the next decade, autopsies failed to confirm that Q wave MI equated to a transmural MI; as a result, in the 1990s, ST segment elevation MI (STEMI) and non-STEMI (NSTEMI) were adopted as the preferred terminology. This new nomenclature would define MI by the ECG changes seen. STEMI was defined as an MI with ST segment elevations in two contiguous leads on ECG (criteria differed somewhat depending on which leads were involved) that most often had complete occlusion of an epicardial coronary artery. Conversely, an NSTEMI was defined as ST depressions or other ECG ischemic changes not meeting the criteria for STEMI. Angiographic studies, some from our own group, indicated that non-Q wave MI (similar to NSTEMI) could result from a total occlusion of a small branch, a total occlusion followed by spontaneous opening (reperfusion) of a large artery, or collateral blood flow from another territory lessening the effects of total occlusion ^3^. In the latter two instances, the amount of necrosis was such that ST elevation did not occur or perhaps was transient.

In 2007, a consensus statement was released by the major American and European cardiac societies with a universal definition of MI. This definition expanded on the previous nomenclature to include lab tests and clinical history. MI became defined as an event with the rise or fall (or both) in a blood test sensitive to heart muscle damage (troponin I or T) along with clinical evidence for a diagnosis of AMI as outlined above. With this universal definition, many causes of NSTEMI did not necessarily require a thrombus in an epicedial artery.

## Pathogenesis of myocardial infarction and the role of thrombosis

The role of thrombosis as a cause of AMI was debated for decades in the 20th century until the 1970s, when it was clearly established as the cause of nearly all AMIs seen at autopsy and most large AMIs presenting clinically ^4, 5^ ( Table 1). Atherosclerosis with subsequent inflammation is the most common and most important driver of thrombosis. The cardinal feature of atherosclerosis is endothelial dysfunction. Atherosclerosis is a chronic inflammatory process of the inner wall (intima) of moderate and large-sized arteries and involves vascular endothelial cells, monocytes, macrophages, T lymphocytes, vascular smooth muscle cells, lipids, and platelets. Atherosclerotic lesions begin as intimal thickening in the coronary artery walls or as fatty streaks. Some progress over time to either thick fibrous-capped or thin fibrous-capped atheromas with a lipid-laden core. Atherosclerotic lesions are prone to acute progression through either asymptomatic thrombosis or intraplaque hemorrhage.

**Table 1.** Coronary thrombosis and acute myocardial infarction: a historical perspective.

• 1910: Obrastzowo and Straschesko described clinical features of AMI • 1912: J. Herrick showed that coronary thrombosis was not invariably fatal • During the next 50 to 60 years, there was controversy as to the cause of AMI • 1966: Constantinitis *et al*. – autopsy of AMI in 16 patients showed that thrombus occurs at the site of plaque fissuring and was the primary event leading to myocardial infarction • 1972: Roberts *et al*. – thrombosis was secondary to AMI and not the primary event leading to AMI • 1974: Chandler *et al*. – National Institutes of Health workshop concluded that thrombosis is the primary event leading to AMI • 1976: Chazov *et al*. – intracoronary steptokinase used in two patients with AMI • 1979: Rentrop *et al*. – intracoronary steptokinase used in five patients with AMI • 1980: DeWood *et al*. conclusively showed that thrombosis is the primary event, with 84% demonstrating total occlusion at angiography in less than 4 hours after evolving transmural AMI • 1980s: Davies, Falk, and others – plaque disruption/erosion at thrombotic site was demonstrated routinely at autopsy in fatal AMI/ sudden cardiac death

AMI, acute myocardial infarction.

Atherosclerosis begins when low-density lipoprotein (LDL) is taken up into the intima and oxidized, resulting in a cascade of inflammatory cytokine, enzyme, and cell adhesion molecule production. This process results in the attraction of T lymphocytes and monocytes into the subintimal space. The accumulation of oxidized LDL further damages the endothelial cells and results in more cytokine- and oxygen-derived free radical production into the subintimal space. Oxidized LDL is subsequently ingested by monocyte-derived macrophages and transformed into foam cells. Over time, smooth muscle cells migrate from the media to the intima and lipid accumulates under a fibrous cap composed of vascular smooth muscle cells, elastin, and collagen. Furthermore, low-grade inflammation as epitomized by an elevated C-reactive protein, independent of LDL levels, has been shown to contribute to myocardial events and hence thought to contribute to the formation and progression of atherosclerotic disease ^6^. As previously mentioned, inflammatory cells such as macrophages and T lymphocytes play a direct role in the formation and destabilization of atherosclerotic plaques. Inflammation also indirectly activates the intrinsic and extrinsic clotting cascades, further contributing to atherosclerotic plaque formation and destabilization ^7^.

The thin-cap fibroatheroma (TCFA), which has a lipid-rich, necrotic core regarded by some as a “vulnerable plaque”, can rupture suddenly because of macrophage infiltration and matrix degradation of the fibrous cap. This results in a cascade of platelet aggregation and thrombus formation that can lead to myocardial ischemia distally or subsequent infarction or both.

Conversely, plaque erosion, another cause of coronary thrombosis that occurs less frequently than plaque rupture, can occur in a lesion rich in proteoglycans and smooth muscle cells but not necessarily in one that is lipid rich. The thrombus here originates from a defect in the endothelial layer that covers the inside wall of all blood vessels. Plaque erosions tend to have fewer inflammatory cells as compared with plaque ruptures. The third mechanism of thrombus formation, which is infrequent (probably seen in <10% of cases), occurs when a calcified nodule protrudes through the thin fibrous cap and results in the platelet aggregation and thrombus formation ^8^. These post-mortem observations were confirmed *in vivo* initially by coronary angiography performed during an acute transmural infarction which confirmed the primary importance of thrombosis as the cause of AMI ^5^ and later by intravascular devices such as optical coherence tomography performed just prior to coronary stent implantation during the acute event ^9^. These intravascular devices can define plaque types associated with AMI.

## Non-ST segment elevation myocardial infarction

Multiple mechanisms as referred to above can cause NSTEMI. Thrombosis is a frequent, but not universal, etiology in NSTEMI, although in this situation, for a multitude of potential reasons, the amount of necrosis is usually less than that in STEMI. All of the above (STEMI or NSTEMI), where a culprit lesion with a presumed thrombus is present in an epicardial artery leading to AMI, are referred to as a type 1 MI. Other types of AMI include a type 2 MI (supply demand mismatch from any process that alters this balance including tachyarrhythmias, extreme swings in blood pressure, and so on), post-percutaneous coronary intervention (post-PCI) (stent implantation), which is a type 4a MI by the universal definition, and post-coronary artery bypass surgery, which is type 5 by the universal definition. Another rare type of AMI is type 3 MI, which occurs when a patient dies from an acute coronary occlusion but no cardiac enzyme marker was obtained prior to the patient’s death or was obtained too early to show a positive value. Some of these causes are discussed below.

It is important to keep in mind that a troponin elevation alone does not necessarily indicate MI unless the appropriate clinical evidence is present. Furthermore, although troponin is sensitive for myocardial injury, it is not specific for AMI. As demonstrated by Javed *et al*., about two-thirds of the time with a sensitive assay (upper reference level of 0.04 ng/dL), a troponin elevation did not meet criteria for MI by the universal definition ^10^.

## Myocardial infarction due to causes other than atherosclerosis

The etiology of MI is not limited to atherosclerosis. Among its causes, there are several diverse etiologies ( Table 2). Coronary artery embolization is a rare cause. The emboli can arise from the left atrium as a consequence of atrial fibrillation or from clots in the left ventricle as a consequence of ventricular aneurysms or severely poor left ventricle systolic function or from prosthetic valves or infected native heart valves. Systemic hypotension, as a result of any etiology of shock, can result in global myocardial ischemia and subsequent infarction. Increased oxygen demand (such as in situations of severe anemia, tachyarrhythmias, or hyperthyroidism), especially in patients with moderate epicardial coronary artery stenosis, can result in significant ischemia and subsequent infarction if not corrected.

**Table 2.** Causes of acute myocardial infarction without coronary atherosclerosis.

• Coronary artery disease other than atherosclerosis (for example, Kawasaki’s syndrome) • Trauma to coronary arteries • Spontaneous coronary dissection • Coronary mural thickening with metabolic disease or intimal proliferation (for example, amyloid and Fabry’s disease) • Other causes of luminal narrowing (for example, spasm) • Coronary emboli • Congenital coronary anomalies • Supply–demand mismatch • *In situ* thrombosis • Other (for example, contusion, complications of angiography, Takotsubo, Takayasu’s arteritis, and giant cell arteritis)

Spontaneous coronary artery dissection is becoming a more recognized etiology of AMI. This occurs when an abrupt and sudden tear occurs in the wall of the coronary artery, resulting in decreased blood flow distally by either thrombus formation or obstructive hematoma formation. It is more common in younger patients and women, and the incidence is higher during pregnancy. Coronary spasm, either idiopathic or drug-induced such as in cocaine use, results in MI as well by decreasing blood supply to the heart muscle. Furthermore, Takayasu’s arteritis and giant cell arteritis have been reported to cause MI as well.

## From the late 1970s to the present

Thrombolytic therapy to dissolve intracoronary thrombus revolutionized the treatment of acute STEMI in the late 1970s. The therapy was first applied directly into the affected coronary artery and then later infused intravenously, providing a mechanism to limit infarct size by opening the infarct artery, restore flow to the muscle, and reduce mortality. In the 1980s, balloon angioplasty was introduced as another method for opening occluded vessels and later the use of a stent became the preferred non-surgical methodology. These types of catheter interventions are generally referred to as PCIs. Randomized trials and registries taught us the importance of rapid reperfusion since “time was muscle”. Our goals of therapy (short- and long-term) included not only rapid reperfusion of the infarct artery but keeping the vessel open with appropriate adjunctive anti-platelet agents, prescribing statins to lower LDL cholesterol and other meds to improve healing of the vessel wall and the myocardium, thus reducing the incidence of other post-infarction complications, including arrhythmias and heart failure ( Table 3). It should be noted that there has also been a trend toward a reduction in the incidence of STEMI over the last several years and this is likely attributable to better preventive strategies, including the use of statins and reduced prevalence of cigarette smoking ^11^.

**Table 3.** Common complications of ST segment elevation myocardial infarction.

• Congestive heart failure • Cardiogenic shock • Tachyarrhythmias • Bradyarrhythmias • Heart block • Pericarditis • Bleeding • Mechanical complications • Death

Appropriate reperfusion therapy, particularly percutaneous coronary intervention, has decreased all complications except bleeding.

Today, the preferred acute management strategy of STEMI is PCI of the infarct lesion if the patient is in a hospital with these capabilities. Otherwise, thrombolytic agents are still used when PCI cannot be performed rapidly after a patient’s presentation, usually because the patient is admitted to a non-PCI-performing hospital ^12^. Typically, thrombolytics are used only within 6 to 12 hours after the onset of symptoms, and the most myocardial salvaging occurs when the agent is given within a few hours of symptom onset. Following thrombolytic intervention, patients are often transferred to a PCI-capable hospital for angiography and possible PCI of the infarct artery. This is needed since there is usually a severe residual narrowing of the infarct-related lesion after the thrombus is partially dissolved and a stent is needed to maximize opening of the artery.

Reperfusion therapy has reduced long-term complications of infarction, including mortality, by as much as 50 to 70%. No longer are most patients condemned to weeks of bed rest and limited activity afterwards. With early reperfusion (usually defined as less than 3 to 6 hours after symptom onset) and the appropriate guideline-directed medical and lifestyle therapies, most patients are discharged within 2 to 3 days of their infarction and can resume normal or near normal lives. However, there are exceptions. Mechanical complications such as rupture of either a papillary muscle head with severe mitral regurgitation or the interventricular septum are usually surgical emergencies. Severe triple-vessel or left main disease is another area where a coronary artery bypass graft (CABG) should be considered, although each case must be individualized. Opening the infarct vessel with PCI followed by staged intervention of the other blockages (either CABG or stenting) represents another option.

A continued problem is the patient with an acute infarction who develops cardiogenic shock unrelated to mechanical complications. This complicates up to 5 to 7% of cases. In-hospital mortality even with rapid reperfusion strategies (usually PCI and occasionally CABG) is at least 40%. Although successful reperfusion reduces mortality versus failed or no reperfusion, there is still the need to significantly improve outcomes ^13^. Other than trying to shorten the time to reperfusion, which in itself is complex as the delays are multifactorial including the patient arriving late to the hospital after symptom onset (several hours to days), new approaches, including the early use of ventricular support to unload the left ventricle and give it time to recover, are being evaluated.

In NSTEMI, our guidelines recommend early risk stratification to help decide downstream management ^14^. Those in a higher risk category usually pursue an invasive strategy (angiography followed by PCI or CABG, if indicated) along with optimal medical therapy. Those at lower risk are generally managed conservatively. In truth, the vast majority will undergo coronary angiography during hospitalization either invasively or non-invasively with computed tomographic angiography. A conundrum often revolves around interpretation of the elevated troponin as referred to previously. Does it represent MI or is it a non-specific indicator of myocardial injury/necrosis? This elevation is frequently seen in patients with worsening heart failure, renal failure, and so on but does not meet the criteria as outlined in the universal definition of MI. Those individuals do not mandate coronary angiography.

It also remains unclear how to definitely diagnose and treat a type 2 MI. Other than treating the underlying cause of the supply–demand mismatch (that is, hypotension and tachycardia), do all patients require identification of their coronary anatomy and the same acute management strategy of a type 1 MI? Until more studies become available, one needs to individualize therapy on the basis of the risk profile and presentation.

## Vulnerable plaques/vulnerable patients

Vulnerable plaque is usually defined as a plaque prone to thrombosis and a future acute coronary event (AMI or sudden coronary death and occasionally unstable angina) ^15^. In most instances (>90%), the underlying pathophysiology is either plaque rupture or plaque erosion with a superimposed thrombus partially or totally occluding the lumen of the artery. When plaque rupture is the cause which accounts for the majority of STEMIs and a large percentage of sudden coronary deaths, the plaque responsible is, as previously discussed, a TCFA with a large lipid-rich, necrotic core, inflamed, and possessing a thin fibrous cap (<65 μm). The tear in the fibrous cap leads to the formation of a platelet-rich white thrombus at the site of rupture followed by a red cell and fibrin red thrombus if the artery becomes totally occluded. Plaque erosion accounts for about one-third of STEMIs and possibly a higher percentage of NSTEMIs. There has been great interest in attempting to identify these vulnerable plaques (mainly the TCFA) prior to a future coronary event with the idea of modifying the presumed MI culprit with a stent and thus preventing the adverse event from occurring ^16^. There are theoretical reasons supporting and refuting this approach, and trials examining this issue are ongoing. However, this approach is not validated at present.

If one cannot identify the plaque, can the patient most likely to develop an acute event be found? Of course, we as physicians use risk scores based on well-established factors associated with the presence of coronary artery disease (CAD). These are the so-called risk factors such as high blood pressure, high cholesterol, diabetes, and smoking which can be used to categorize patients into low, intermediate, and high risk. Although it makes intuitive sense that the highest-risk individuals are most likely to develop an adverse event, most initial events on follow-up arise from the lower-risk individuals, as high-risk individuals represent a small portion of the population (<10%) ^17^. At present, there is no consensus about how to find these lower-risk patients in primary prevention, while patients in secondary prevention are all treated with appropriate guideline-directed therapy, as they are considered high-risk. Should we be more aggressive than the guidelines indicate in primary prevention and treat more patients with medications such as statins in addition to lifestyle changes of a healthy diet and exercise, and at what age should one start? These remain unanswered questions.

## Future directions

We have come a long way over the last several decades in our understanding and treatment of coronary atherosclerosis and its complications. Although we have not considered stroke, many of the same drugs and techniques described above for MI are applicable in its prevention and acute treatment. What are the next steps? Billions of dollars are spent yearly on new drug and other treatment strategies in CAD which are generally applied either in secondary prevention or in high-risk primary prevention. But isn’t that a little late? To significantly reduce the incidence and improve outcomes above those seen with our later therapies as described above, we think the best option is earlier intervention ^18^.

Symptomatic CAD occurs decades after the onset of atherosclerosis. Like an iceberg, it does not rear its head above the water line (or, in CAD management, become symptomatic) until there is a critical mass of ice (a large burden of atherosclerosis). This asymptomatic stage is where our efforts should be concentrated if we want to eliminate a majority of future coronary events. These should include earlier identification and treatment of high blood pressure, eliminating tobacco, and lowering average LDL levels. Earlier identification of atherosclerosis will lead to lower event rates if the proper lifestyle and possibly drug therapies are initiated. In the future, new strategies and risk profiles (possibly with genetic profiling) may help to better identify those at risk. New treatments specifically targeting inflammation might also result in a reduction in events as long as the treatments do not interfere with a patient’s natural immunity and erase any potential benefits. Another possibility for the future is a vaccine against atherosclerosis. Finally, wouldn’t it be a crowning achievement of this century if articles such as this should become more of historic interest rather than a review of current practice?

## Abbreviations

AMI, acute myocardial infarction; CABG, coronary artery bypass graft; CAD, coronary artery disease; ECG, electrocardiogram; LDL, low-density lipoprotein; MI, myocardial infarction, NSTEMI, non-ST segment elevation myocardial infarction; PCI, percutaneous coronary intervention; STEMI, ST segment elevation myocardial infarction; TCFA, thin-cap fibroatheroma
